# FOXM1 evokes 5-fluorouracil resistance in colorectal cancer depending on ABCC10

**DOI:** 10.18632/oncotarget.14351

**Published:** 2016-12-29

**Authors:** Tao Xie, Jian Geng, Ye Wang, Liya Wang, Mengxi Huang, Jing Chen, Kai Zhang, Lijun Xue, Xiaobei Liu, Xiaobei Mao, Yanan Chen, Qian Wang, Tingting Dai, Lili Ren, Hongju Yu, Rui Wang, Longbang Chen, Cheng Chen, Xiaoyuan Chu

**Affiliations:** ^1^ Department of Medical Oncology, Jinling Hospital, Nanjing Clinical School of Southern Medical University, Nanjing 210002, China; ^2^ Department of Medical Oncology, Jinling Hospital, School of Medicine, Nanjing University, Nanjing 210002, China

**Keywords:** colorectal cancer, FOXM1, 5-FU, chemoresistance

## Abstract

5-Fluorouracil (5-FU) is the most commonly used chemotherapeutic agent for colorectal cancer (CRC). However, frequently occurred 5-FU resistance poses a great challenge in the clinic. Elucidating the underlying mechanisms and developing effective strategies against 5-FU resistance are highly desired. Here we identified the upregulation of FOXM1 in 5-FU nonresponsive CRC patients by gene expression profile analysis and 5-FU-resistant CRC cells by qRT-PCR assay. Silencing of FOXM1 promoted the sensitivity of CRC cells to 5-FU by enhancing cell apoptosis, while overexpression of FOXM1 conferred CRC cells with 5-FU resistance both *in vitro* and *in vivo*. Furthermore, we showed that genetic and pharmacological inhibition of FOXM1 resensitized resistant CRC cells to 5-FU treatment. Mechanistically, FOXM1 promoted the transcription of ABCC10 by directly binding to its promoter region. Notably, treatment with ABCC10 inhibitor reversed FOXM1-induced resistance to 5-FU *in vivo*. Clinical investigation revealed that the levels of FOXM1 and ABCC10 were positively correlated in CRC tissues. Therefore, FOXM1 promotes 5-FU resistance by upregulating ABCC10, suggesting that FOXM1/ABCC10 axis may serve as a potential therapeutic target for 5-FU resistance in CRC patients.

## INTRODUCTION

Colorectal cancer (CRC) is one of the most prevalent malignancy and the third leading cause of cancer-related death worldwide [1]. Despite therapeutic strategies involving surgical resection, radiotherapy and chemotherapy, the survival rate of CRC patients remains unsatisfactory [2]. 5-Fluorouracil (5-FU) is one of the main component of adjuvant and palliative chemotherapy for CRC patients [3, 4]. It elicits cytotoxicity by inhibiting the nucleotide synthetic enzyme thymidylate synthase (TYMS), as well as by incorporating fluoronucleotides into RNA and DNA [5]. It has shown that a fraction of CRC patients are inherently refractory to 5-FU-based chemotherapy, and most of the remaining patients will acquire chemoresistance after treatment, posing a major challenge for therapeutic efficiency in the clinic. Thus, it is urgently needed to elucidate the underlying mechanisms and discover potential targets for the treatment of CRC patients with 5-FU resistance.

FOXM1 is a key transcription factor in cell cycle progression [6], DNA replication [7], angiogenesis [8], metastasis [9] and drug resistance [10], which belongs to a large Forkhead Box family that share a conserved winged-helix DNA-binding domain [11]. FOXM1 has been reported to be highly expressed in a variety of human cancers, including hepatocellular carcinoma, glioma, gastric cancer, lung caner, cervical cancer and CRC [8, 12–16]. Our previous study has shown that FOXM1 expression correlated with CRC metastasis and predicts poor prognosis of CRC patients [17]. Several studies have demonstrated that FOXM1 involves in the resistance to cisplatin, paclitaxel, docetaxel and epirubicin, indicating that FOXM1 plays important roles in drug resistance [10, 18–24]. However, the role and underlying mechanism of FOXM1 in the 5-FU resistance of CRC remain to be elucidated. In this study, we investigate the contribution of FOXM1 to 5-FU resistance and explore potential therapeutic implications for 5-FU-resistant CRC patients.

## RESULTS

### FOXM1 is upregulated in 5-FU resistant CRC

**Figure 1:**
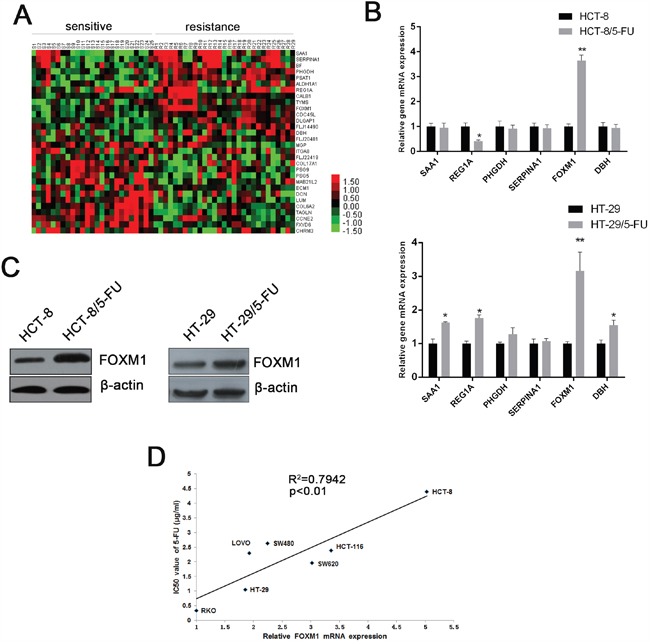
FOXM1 is upregulated in 5-FU resistant CRC **A**. Heatmap of gene expression levels of top 15 upregulated genes and top 15 downregulated genes in resistant CRC tissues compared with sensitive CRC tissues. **B**. qRT-PCR assay of the top 6 upregulated genes in HCT-8 and HCT-8/5-FU cells or HT-29 and HT-29/5-FU cells (n=3). β-actin serves as an internal reference. **C**. Western blot assay of FOXM1 in HCT-8 and HCT-8/5-FU cells or HT-29 and HT-29/5-FU cells. β-actin serves as an internal reference. **D**. Pearson correlation analysis of FOXM1 expression and 5-FU IC50 value in CRC cell lines. Statistical significance was determined by Student's t test. *p<0.05, **p<0.01.

In order to explore genes involving in 5-FU resistance in CRC, we analyzed the gene expression profile of pre-therapy biopsies of CRC patients who received 5-FU-based chemotherapy afterward (Supplementary Table 1) [25]. Heatmap showed distinction between chemo-sensitive and resistant patients (Figure 1A). Six of the most differentially expressed genes (fold change >1. 8) were subjected to validation by qRT-PCR in 5-FU sensitive and resistant CRC cells (Supplementary Figure 1A). Among these genes, FOXM1 was the most notably upregulated transcript in 5-FU-resistant CRC cells (Figure 1B). Western blot analysis further verified the upregulation of FOXM1 in resistant cells at protein level (Figure 1C). We next detected FOXM1 expression and the IC50 value of 5-FU in various CRC cell lines (RKO, HCT-116, HT-29, LOVO, SW620, HCT-8, SW480) (Supplementary Figure 1B and 1C). As shown in Figure 1D, the expression of FOXM1 was positively correlated with the IC50 value of 5-FU in CRC cell lines. In addition, those cells with low FOXM1 levels had increased FOXM1 expression after exposure to 5-FU (Supplementary Figure 1D). Altogether, these data indicate that FOXM1 is highly expressed in 5-FU resistant CRCs.

### Silencing of FOXM1 restores the sensitivity of CRC to 5-FU

**Figure 2:**
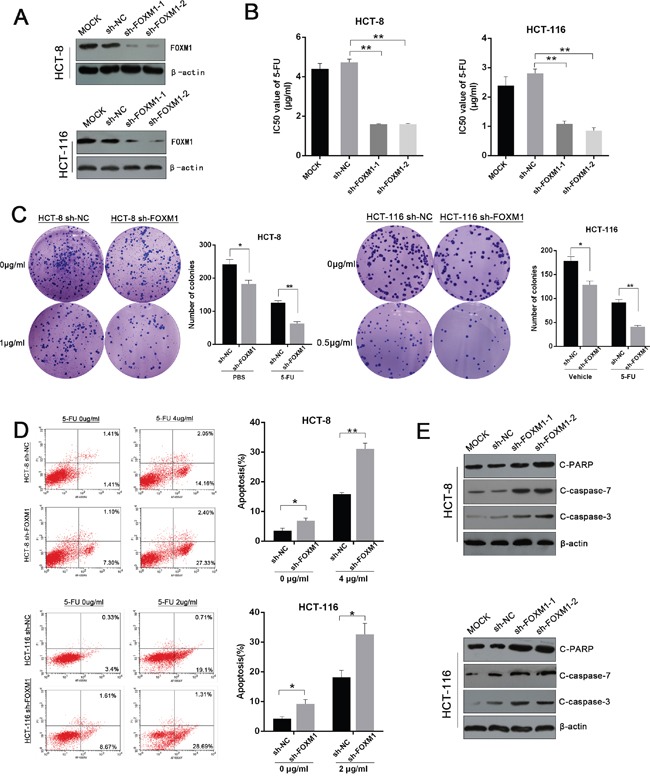
Silencing of FOXM1 restores the sensitivity of CRC to 5-FU **A**. Western blot assay of FOXM1 in knockdown and control CRC cells. Upper: HCT-8 cells. Lower: HCT-116 cells. **B**. IC50 values of 5-FU in FOXM1 knockdown and control cells determined by CCK-8 assay (n=3). **C**. Colony formation of FOXM1 knockdown and control CRC cells treated with indicated concentrations of 5-FU (n=3). Representative images and average number of colonies are shown. **D**. Flow cytometric analysis of apoptotic cells in FOXM1 knockdown and control CRC cells treated with indicated concentrations of 5-FU (n=3). Representative images (left) and average percentage of apoptotic cells (right) are shown. **E**. Western blot assay of cleaved PARP, cleaved caspase-3 and cleaved caspase-7 in FOXM1 knockdown HCT-8 and HCT-116 cells upon 5-FU treatment (4 μg/ml and 2 μg/ml, respectively). Statistical significance was determined by Student's t test. *p<0.05, **p<0.01.

To determine the functional role of FOXM1 in 5-FU resistance, we stably knocked down FOXM1 expression in CRC cells by two independent lentiviral-mediated short hairpin RNAs (shRNAs) (Figure 2A, Supplementary Figure 2A and Supplementary Table 2). Compared with sh-NC control, the IC50 values of 5-FU were significantly reduced upon FOXM1 depletion (p<0. 01) (Figure 2B). Colony formation assay revealed that silencing of FOXM1 reduced cell viability under 5-FU treatment (Figure 2C). Although knockdown of FOXM1 alone could inhibit cell proliferation, the difference between two groups were more significant under 5-FU treatment (Supplementary Figure 2B). Additionally, knockdown of FOXM1 elicited enhanced cell apoptosis upon 5-FU exposure (Figure 2D). Consistently, western blot analysis showed increased cleaved PARP, cleaved caspase-3 and cleaved caspase-7 in 5-FU-treated FOXM1 knockdown cells (Figure 2E), suggesting that silencing FOXM1 induced cell death through caspase-dependent apoptotic pathways. Collectively, these results indicate that suppression of FOXM1 sensitizes CRC cells to 5-FU.

### Overexpression of FOXM1 confers 5-FU resistance to CRC cells

**Figure 3:**
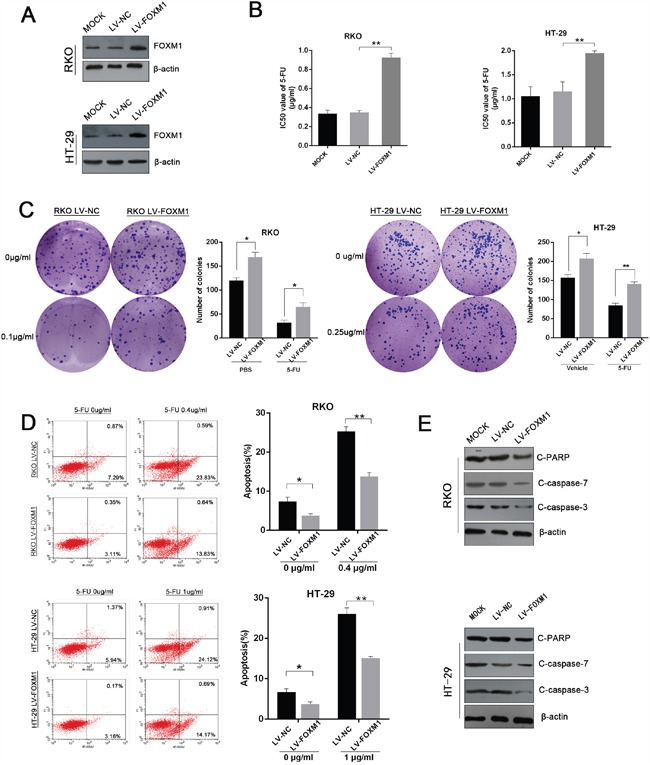
Overexpression of FOXM1 confers 5-FU resistance to CRC cells **A**. Western blot assay of FOXM1 in overexpression and control CRC cells. Upper: RKO cells. Lower: HT-29 cells. **B**. IC50 values of 5-FU in FOXM1 overexpression and control cells determined by CCK-8 assay (n=3). **C**. Colony formation of FOXM1 overexpression and control CRC cells treated with indicated concentrations of 5-FU (n=3). Representative images and average number of colonies are shown. **D**. Flow cytometric analysis of FOXM1 overexpression and control CRC cells treated with indicated concentrations of 5-FU (n=3). Representative images (left) and average percentage of apoptotic cells (right) are shown **E**. Western blot assay of cleaved PARP, cleaved caspase-3, cleaved caspase-7 in FOXM1 overexpressing RKO and HT-29 cells upon 5-FU treatment (0.1 μg/ml and 0.4 μg/ml, respectively). Statistical significance was determined by Student's t test. *p<0.05, **p<0.01.

We next examined whether ectopic expression of FOXM1 could confer 5-FU resistance to CRC cells. RKO and HT-29 were selected and infected with FOXM1 overexpressing lentivirus (Figure 3A and Supplementary Figure 3A). As expected, overexpression of FOXM1 increased 5-FU IC50 in both cells (Figure 3B). FOXM1 overexpressing cells also formed more clones than control cells upon 5-FU treatment (Figure 3C and Supplementary Figure 3B). Furthermore, enforced expression of FOXM1 resulted in a dramatic decrease of apoptosis as illustrated by flow cytometry and western blot (Figure 3D and 3E), indicating that overexpression of FOXM1 could confer 5-FU resistance to CRC cells.

### FOXM1 enhances CRC resistance to 5-FU *in vivo*

**Figure 4:**
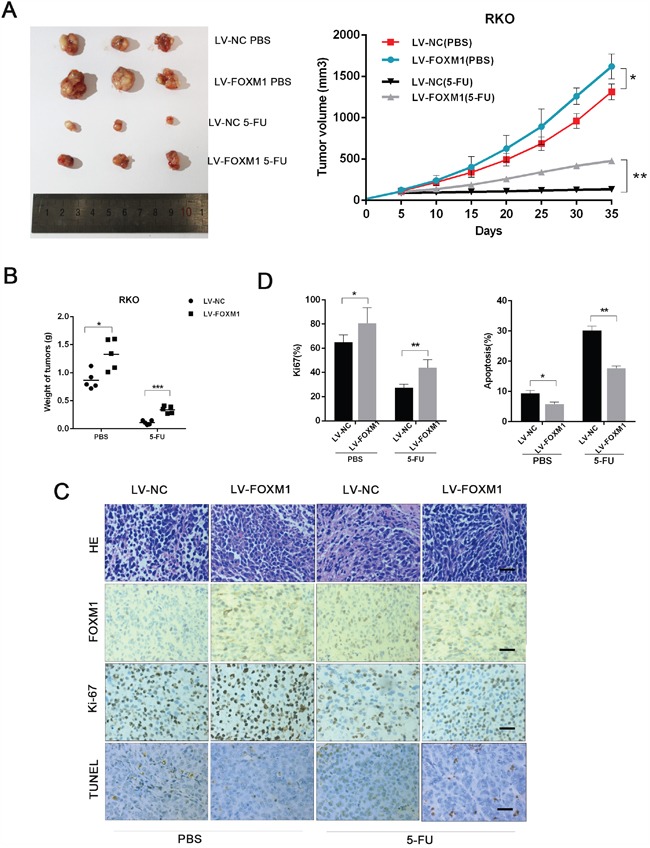
FOXM1 enhances CRC resistance to 5-FU in vivo **A**. Nude mice were subcutaneously xenografted with FOXM1 overexpression or control cells (5×106) and intraperitoneally injected with 5-FU (5 mg/kg) every three days. Representative images of tumors and tumor volumes are shown. **B**. Average weight of tumors derived from each group are shown. **C**. H&E and immunostaining of FOXM1, Ki-67 and TUNEL in tumor sections (scale bar, 25 μm). **D**. Average percentage of Ki-67 positive cells and apoptotic cells in xenografts from each group. Statistical significance was determined by Student's t test. *p<0.05, **p<0.01.

To investigate the effect of FOXM1 on the 5-FU sensitivity of CRC *in vivo*, FOXM1 overexpressing RKO cells were subcutaneously transplanted into nude mice, followed by 5-FU treatment. As shown in Figure 4A and 4B, a difference in tumor growth was observed between FOXM1 overexpressing and control CRC cells, whereas the difference was more significant upon 5-FU treatment, indicating that FOXM1 conferred 5-FU tolerance to CRC cells. Moreover, immunohistochemistry (IHC) analysis of xenografted tumors showed that RKO cells with FOXM1 overexpression had a more significant high level of cell proliferation (Ki-67) and low level of apoptosis (TUNEL) compared to control group under 5-FU treatment (Figure 4C and 4D). Additionally, compared with vehicle-treated LV-NC xenografts, FOXM1 level was increased in the LV-NC xenografts of 5-FU-treated mice (Supplementary Figure 4A and 4B). Therefore, the *in vivo* data confirm that FOXM1 improves the 5-FU tolerance of CRC cells.

### Genetic and pharmacological inhibition of FOXM1 restores the sensitivity of resistant CRC cells to 5-FU

**Figure 5:**
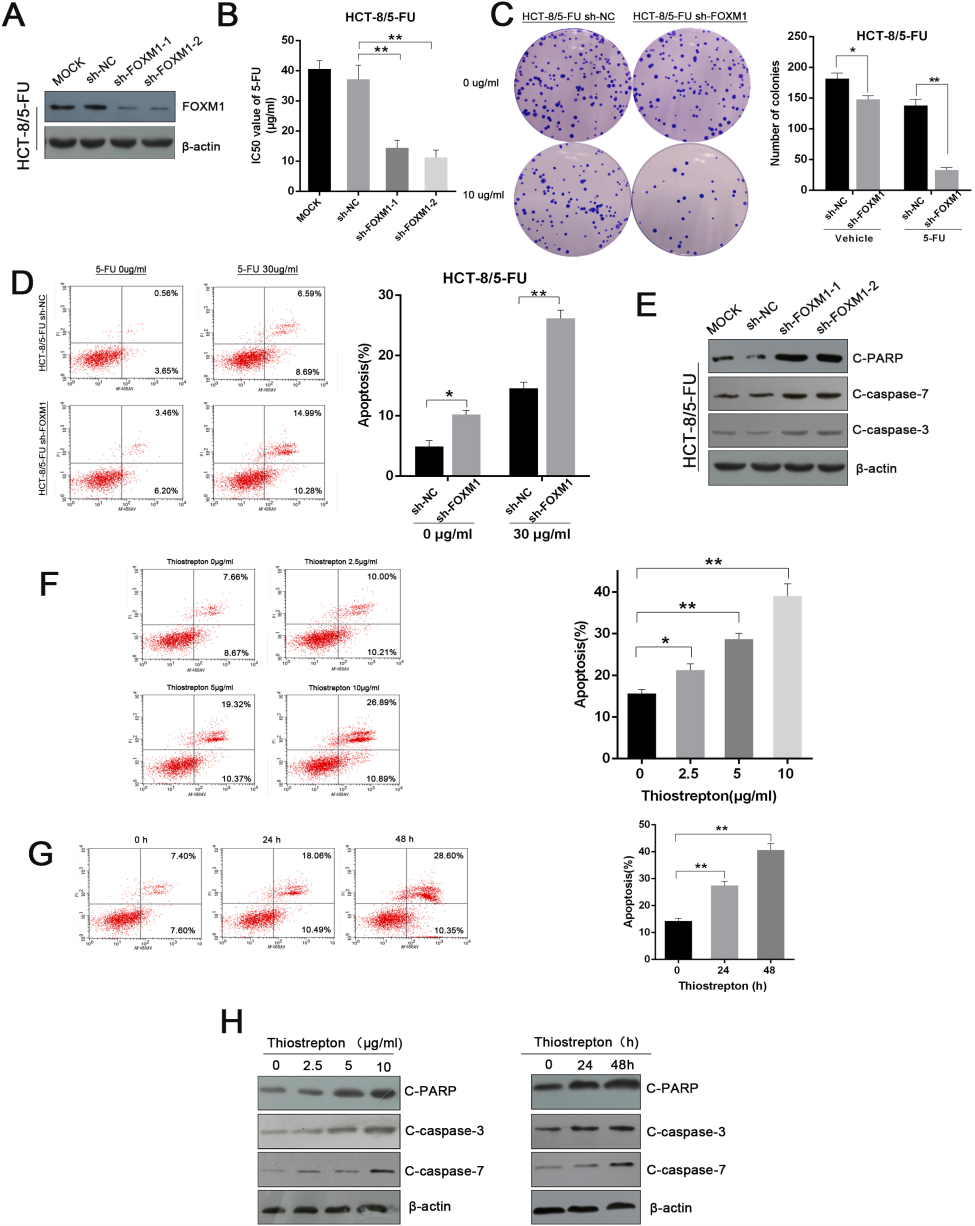
Genetic and pharmacological inhibition of FOXM1 restores the sensitivity of resistant CRC cells to 5-FU **A**. Western blot assay of FOXM1 in knockdown and control 5-FU-resistant HCT-8 cells (HCT-8/5-FU). **B**. IC50 values of 5-FU in FOXM1 knockdown and control cells determined by CCK-8 assay (n=3). **C**. Colony formation of FOXM1 knockdown and control HCT-8/5-FU cells treated with indicated concentrations of 5-FU (n=3). Representative images and average number of colonies are shown. **D**. Flow cytometric analysis of FOXM1 knockdown and control HCT-8/5-FU cells treated with indicated concentrations of 5-FU (n=3). Representative images (left) and average percentage of apoptotic cells (right) are shown **E**. Western blot assay of cleaved PARP, cleaved caspase-3, and cleaved caspase-7 in FOXM1 knockdown HCT-8/5-FU cells upon 5-FU treatment (30 μg/ml). **F**. Flow cytometric analysis of apoptotic cells in HCT-8/5-FU cells treated with 5-FU (30μg/ml) and thiostrepton at indicated concentrations for 24h. **G**. Flow cytometric analysis of apoptotic cells in HCT-8/5-FU cells treated with 5-FU (30 μg/ml) and thiostrepton (5 μg/m) for indicated times. **H**. Western blot assay of cleaved PARP, cleaved caspase-3 and cleaved caspase-7 in HCT-8/5-FU cells upon 5-FU (30 μg/ml) and thiostrepton treatment at indicated concentrations or times. Statistical significance was determined by Student's t test. *p<0.05, **p<0.01.

To further confirm the role of FOXM1 in 5-FU resistance, we silenced FOXM1 in established 5-FU-resistant CRC cells (Figure 5A and Supplementary Figure 5A). As expected, interference of FOXM1 led to decreased IC50, attenuated growth ability and increased apoptosis in resistant cells upon 5-FU treatment (Figure 5B-5E and Supplementary Figure 5B). We also utilized thiostrepton, a selective FOXM1 inhibitor, that reduced FOXM1 expression as previously reported (Supplementary Figure 5C) [26]. Consistently, thiostrepton induced an increased apoptosis in 5-FU-resistant cells in dose-dependent and time-dependent manner (Figure 5F-5H). These genetic and pharmacological data indicate that FOXM1 is critical in the 5-FU resistance of CRC.

### Inhibition of FOXM1 resentisizes resistant CRC to 5-FU *in vivo*

**Figure 6:**
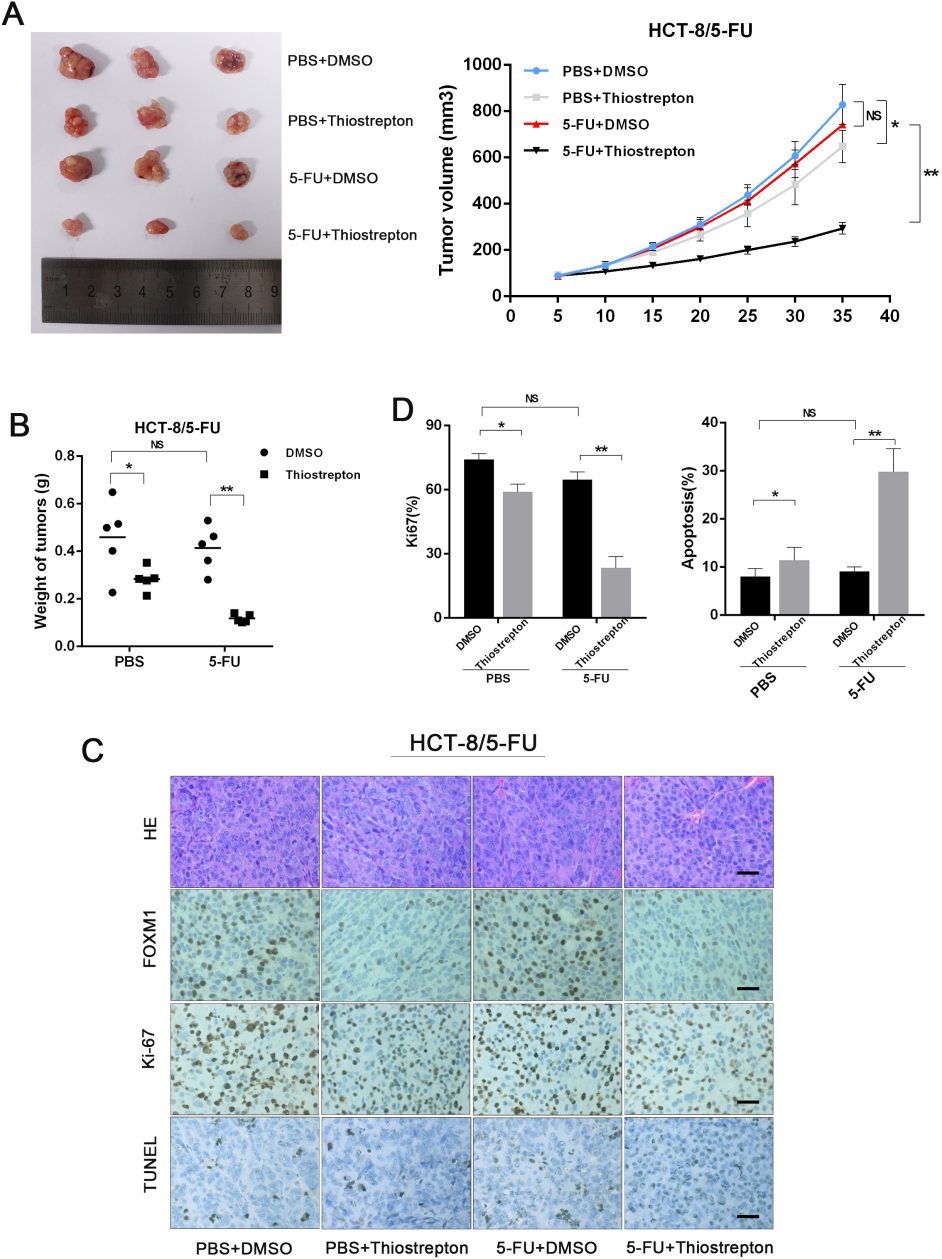
Inhibition of FOXM1 resentisizes resistant CRC to 5-FU in vivo **A**. Nude mice were subcutaneously xenografted with 5-FU resistance CRC cells (HCT-8/5-FU) and intraperitoneally injected with 5-FU (5 mg/kg) along with Thiostrepton (17 mg/kg) every three days. Representative images of tumors and tumor volumes are shown. **B**. Average weight of tumors derived from each group are shown. **C**. H&E and immunostaining of FOXM1, Ki-67 and TUNEL in tumor sections are shown (scale bar, 25 μm). **D**. Average percentage of Ki-67 positive cells and apoptotic cells in xenografts from each group. Statistical significance was determined by Student's t test. *p<0.05, **p<0.01. NS, not significant.

To evaluate the therapeutic potential of FOXM1 in 5-FU-resistant CRC *in vivo*, thiostrepton was intraperitoneally injected into mice xenografted with HCT-8/5-FU cells. As shown in Figure 6A and 6B, thiostrepton restored the sensitivity of HCT8/5-FU xenografts to concurrent 5-FU treatment, whereas 5-FU alone led to no growth inhibition. Consistently, the IHC analysis of xenografts revealed that combined treatment of thiostrepton and 5-FU resulted in decreased cell proliferation (Ki-67) and increased cell apoptosis (TUNEL) compared with 5-FU or thiostrepton treatment alone (Figure 6C and 6D). These data further indicate that inhibition of FOXM1 could restore the response of resistant CRC to 5-FU.

### FOXM1 promotes 5-FU resistance by directly enhancing ABCC10 transcription

**Figure 7:**
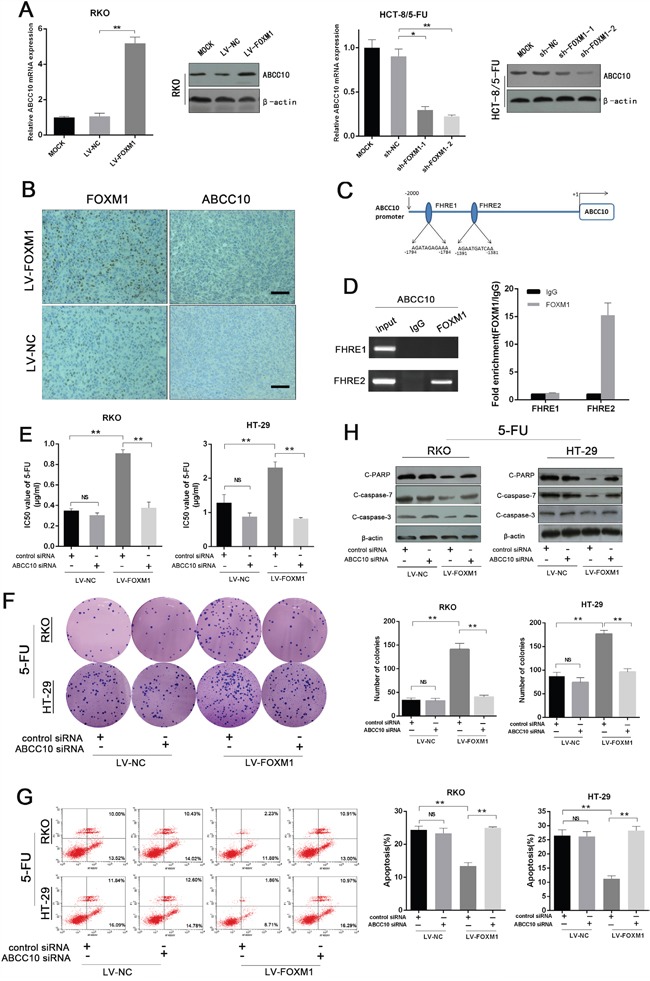
FOXM1 promotes 5-FU resistance by directly enhancing ABCC10 transcription **A**. qRT-PCR and western blot analysis of ABCC10 in FOXM1 overexpressing or knockdown CRC cells. Left: RKO cells. Right: HCT-8/5-FU cells. **B**. Representative immunostaining of FOXM1 and ABCC10 in FOXM1 overexpressing and control xenografted tumors (scale bar, 50 μm). **C**. Putative FOXM1 binding sites on the promoter region of ABCC10. **D**. Upper: gel electrophoresis of PCR products from ChIP assay. Lower: ChIP assay of the enrichment of FOXM1 on ABCC10 promoter relative to IgG in HCT-8/5-FU cells. **E**. IC50 values of 5-FU in FOXM1 overexpression and control cells co-transfected with si-ABCC10 or si-NC as determined by CCK-8 assay. **F**. Colony formation in FOXM1 overexpression and control cells co-transfected with si-ABCC10 or si-NC upon 5-FU treatment (n=3). Representative images and average number of colonies are shown. **G**. Flow cytometric analysis of apoptotic cells in FOXM1 overexpression and control cells co-transfected with si-ABCC10 or si-NC upon 5-FU treatment (n=3). Representative images and average number of colonies are shown. **H**. Western blot assay of cleaved PARP, cleaved caspase-3 and cleaved caspase-7 in FOXM1 overexpressing CRC cells co-transfected with si-ABCC10 or si-NC upon 5-FU treatment. Statistical significance was determined by Student's t test. *p<0.05, **p<0.01. NS, not significant.

We next investigated the underlying mechanism for FOXM1-driven 5-FU resistance. It is well known that 5-FU-metabolizing enzymes and membrane drug transporters are closely related to 5-FU resistance [27–31]. Concerning that FOXM1 functions as a transcription factor, we first screened the promoter regions of genes related to 5-FU metabolism and drug efflux for FOXM1 binding elements. Ten genes were selected out and subjected to qRT-PCR validation in FOXM1 overexpressing and knockdown resistant cells. As shown in Supplementary Figure 6A, the mRNA level of ABCC10 exhibited the most robust change following FOXM1 alteration, which was confirmed by western blot (Figure 7A). Consistently, high expression of ABCC10 was observed in FOXM1 overexpressing xenografts or LV-NC xenografts from 5-FU-treated mice (Figure 7B and Supplementary Figure 6B-6C). Bioinformatic analysis identified two consensus forkhead response elements (FHREs) in the promoter region of ABCC10 (Figure 7C). Moreover, chromatin immunoprecipitation (ChIP) assay confirmed the enrichment of FOXM1 on the promoter region of ABCC10 (Figure 7D), indicating that FOXM1 directly promoted ABCC10 transcription.

Next, we determined the role of ABCC10 in FOXM1-elicited 5-FU resistance of CRC cells. Downregulation of ABCC10 abolished FOXM1-induced resistance to 5-FU, as illustrated by IC50, colony formation assay and apoptosis evaluation (Figure 7E-7H, Supplementary Figure 6D and Supplementary Table 2). Together, these data indicate that FOXM1 promotes 5-FU resistance by upregulating ABCC10 in CRC cells.

### Targeting ABCC10 reverses FOXM1-elicited 5-FU resistance *in vivo*

**Figure 8:**
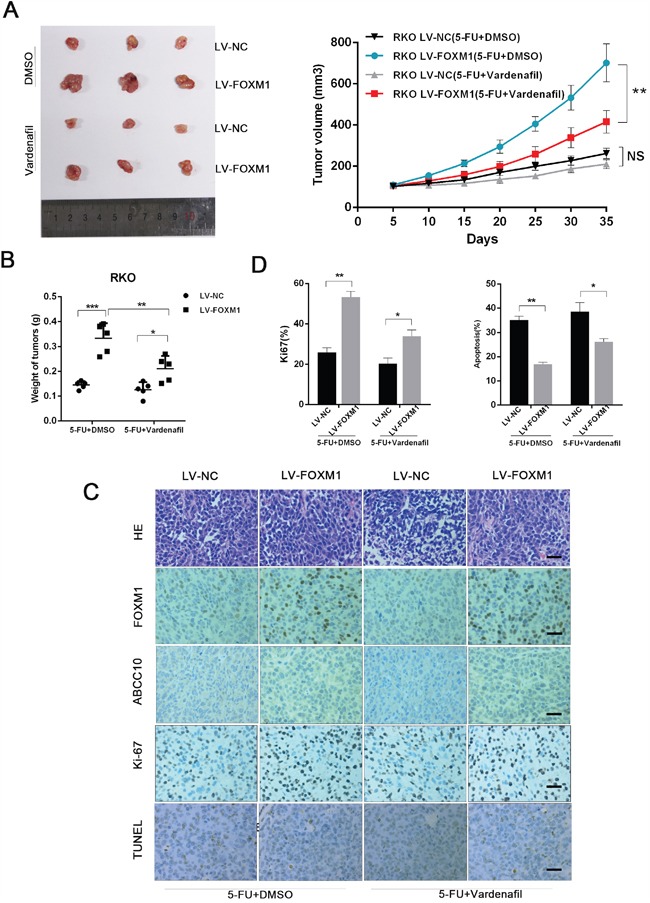
Targeting ABCC10 reverses FOXM1-elicited 5-FU resistance in vivo **A**. Nude mice were subcutaneously xenografted with FOXM1 overexpression or control CRC cells (5×106) and intraperitoneally injected with 5-FU (5 mg/kg) along with vardenafil (22.4 mg/kg) every three days. Representative images of tumors and tumor volumes are shown. **B**. Average weight of tumors derived from each group are shown. **C**. H&E and immunostaining of FOXM1, ABCC10, Ki-67 and TUNEL in tumor sections are shown (scale bar, 25μm). **D**. Average percentage of Ki-67 positive cells and apoptotic cells in xenografts from each group. Statistical significance was determined by Student's t test. *p<0.05, **p<0.01, ***p<0.001. NS, not significant.

As ABCC10 was responsible for FOXM1-mediated 5-FU resistance, vardenafil [32], an ABCC10 inhibitor, was investigated *in vivo*. As show in Figure 8A and 8B, the combination of vardenafil and 5-FU achieved a notable growth suppression on FOXM1 overexressing xenografts, which was refractory to 5-FU treatment alone. Furthermore, IHC analysis of xenografts revealed that concurrent treatment of vardenafil and 5-FU led to decreased cell proliferation (Ki-67) and increased cell apoptosis (TUNEL) compared to 5-FU alone in FOXM1 overexpressing tumors (Figure 8C and 8D). These data suggest that inhibition of ABCC10 reverses FOXM1-mediated 5-FU resistance in CRCs.

### The level of FOXM1 and ABCC10 are correlated in CRC patient tissues

**Figure 9:**
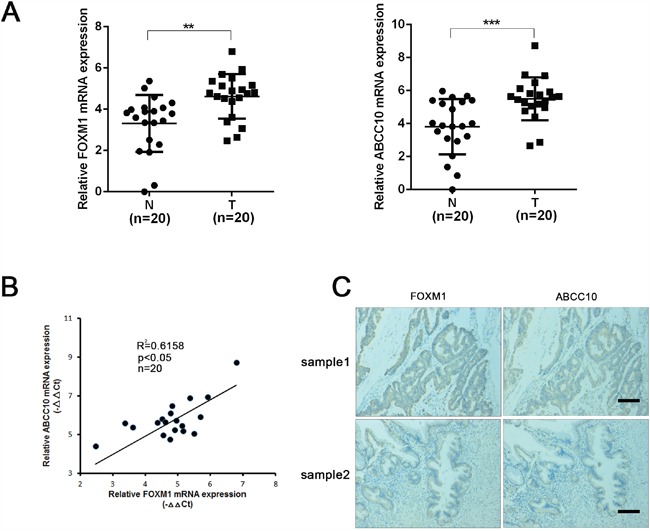
The level of FOXM1 and ABCC10 are correlated in CRC patient tissues **A**. qRT-PCR assay of FOXM1 and ABCC10 in 20 paired CRC and nontumor tissues. The FOXM1 and ABCC10 expression was normalized to β-actin (ΔCt) and compared with the maximum ΔCt. Data were presented as -ΔΔCt. **B**. The bivariate relation between the transcription levels of FOXM1 and ABCC10 in 20 CRC tissues assessed by Pearson's correlation test. **C**. Representative immunostaining of FOXM1 and ABCC10 in consecutive sections of CRC patient tissues (scale bar, 100μm). Statistical significance was determined by Student's t test. *p<0.05, **p<0.01, ***p<0.001.

To extend our findings to human CRC tissues, we investigated the relationship between FOXM1 and ABCC10 expression in tumor tissues of 20 CRC patients. As shown in Figure 9A, the mRNA levels of FOXM1 and ABCC10 were increased in CRC tissues compared to nontumor tissues. Pearson correlation analysis showed that the level of FOXM1 was correlated with that of ABCC10 in tumor tissues (R^2^= 0.6158, p<0. 05) (Figure 9B). Moreover, correlation of FOXM1 and ABCC10 expression was confirmed in consecutive human CRC tissues by IHC analysis (Figure 9C).

## DISCUSSION

Fluorouracil-based chemotherapy is the most common chemotherapy for CRC patients with unresectable or metastatic tumors [33]. However, chemoresistance, either inherent or acquired, is a major challenge in the clinic. It is necessary to explore the mechanisms by which CRC cells acquire 5-FU resistance and develop therapeutic strategies to overcome the resistance. In this study, we found that FOXM1 was upregulated in the CRC tissues of 5-FU nonresponsive patients. Mechanically, FOXM1 promoted 5-FU resistance by directly enhancing the transcription of ABCC10. We also showed that targeting ABCC10 could reverse FOXM1-mediated 5-FU resistance. Therefore, FOXM1/ABCC10 axis was verified to play a crucial role in 5-FU resistance.

Advances in high-throughput technology have yielded massive data of tumor profiles which provides great fortune for cancer study. The application of bioinformatic analysis of the big data has revealed that specific genes with aberrant expression may be critical in cancer development or act as potential biomarkers for cancer diagnosis and prognosis. In an attempt to identify genes that might be implicated with the 5-FU resistance of CRC, raw gene expression data from CRC patients prior to 5-FU-based chemotherapy were collected from GEO database (GSE3964). Comparison of gene expression profile between chemo-sensitive and resistant patients revealed distinct difference. Thymidylate synthase (TYMS), a well-known 5-FU metabolizing enzyme participating in 5-FU resistance [34], appeared in the top 10 upregulated genes in resistance group, proving the reliability of our analysis. Further validation revealed that FOXM1 was the most upregulated transcript in 5-FU resistant CRC cells.

FOXM1 has been reported to play important roles in drug resistance. For instance, FOXM1 confers resistance to paclitaxel by altering microtubule dynamics via promoting Stathmin expression in breast cancer [10]. Depletion of FOXM1 resensitized breast cancer cells to epirubicin-induced cellular senescence [20]. In our previous study, we found that miR-149 is significantly downregulated in 5-FU resistant CRC cells, and restoration of miR-149 could reverse the resistance of CRC cells to 5-FU by directly targeting FOXM1 [35], suggesting the role of FOXM1 in the 5-FU resistance of CRC cells. In the present study, we investigated comprehensively *in vitro* and *in vivo* to elucidate the role of FOXM1 in 5-FU resistance. Overexpression of FOXM1 enhanced cell viabilty and protected cells from 5-FU induced apoptosis, conferring 5-FU resistance to CRC cells both *in vitro* and *in vivo*. Vice versa, interference of FOXM1 restored the sensitivity of resistant cells to 5-FU. Thiostrepton, a specific inhibitor of FOXM1, functions by suppressing FOXM1 expression and also impeding FOXM1 to interact with genomic target sites [26, 36, 37]. Our data showed that thiostrepton resensitized resistant cells to 5-FU treatment in a dose- and time-dependent manner. Thus, we propose that FOXM1-targeted therapy could be used as a promising therapeutic strategy for 5-FU-resistant CRC.

Numerous mechanisms account for chemotherapy resistance, such as elevated drug efflux, increased DNA damage repair, activation of detoxifying systems, and evasion of drug-induced apoptosis etc [38–41]. FOXM1 has also been shown to serve as a negative regulator of senescence, which was associated with chemoresistance [20, 42]. Here, by detecting the expression of a panel of chemoresistance-associated genes, we found that ABCC10 exhibited concomitant changes along with FOXM1 alteration. Furthermore, we demonstrated that FOXM1 promoted ABCC10 expression by directly binding to its promoter region. ABCC10 is a member of ABC transporter family, which play important roles in drug resistance by effluxing anticancer agents outside of cancer cells [43]. ABCC10 has been reported to confer resistance to vinorelbine and paclitaxel in non-small cell lung cancer [44, 45] and salivary gland adenocarcinoma [39]. Our results showed that silencing of ABCC10 reversed FOXM1-elicited 5-FU resistance, indicating that FOXM1-mediated 5-FU resistance in CRC is, at least in large part, dependent on ABCC10. Moreover, we provide evidence for using vardenafil, an inhibitor of ABCC10, to restore 5-FU response in FOXM1-mediated resistance *in vivo*. Vardenafil has already been aproved in erectile dysfunction patients in the clinic [46]. Therefore, targeting ABCC10 provides a strategy for sensitizing FOXM1-abundant CRC to 5-FU.

In conclusion, our results demonstrate that FOXM1/ABCC10 axis contributes to the 5-FU resistance of CRC, and may serve as potential therapeutic targets to overcome 5-FU resistance.

## MATERIALS AND METHODS

### Microarray data analysis

The raw gene profile data of colorectal cancer patients were downloaded from GEO data repository (GSE3964). All samples were collected prior to exposure to combined chemotherapy of folinic acid, 5-FU and irinotecan. Patients were divided into chemo-sensitive group and resistant group according to their initial response to the chemotherapy. Limma package was used for the statistical analysis with an empirical Bayes method. Heatamp was contructed with Cluster 3.0. Raw data were listed in Supplementary Table 1.

### Patient samples

CRC tissues and corresponding nontumor tissues were obtained from 20 patients who received colectomy in Jinling Hospital, Medical School of Nanjing University between March 2012 and March 2014. The samples were frozened immediately in liquid nitrogen. All clinical samples were collected with written informed consent from patients in our department, and the ethical approval was granted from the Review Board of Hospital Ethics Committee (Jingling Hospital, Nanjing University).

### Mice xenograft models

BALB/cathymic nude mice (male, 6 weeks) were provided by the department of comparative medicine of Jinling hospital. Approximately 5×10^6^ RKO cells were subcutaneously implanted into the posterior flank of nude mice. Xenograft size was measured every 3 days and calculated by using the equation V (mm^3^)= (length × width^2^)/2. When the tumors grew to 100 mm^3^, 5-FU was given through intraperitoneal injection at a concentration of 5 mg/kg every three days. For therapeutic experiments, 5-FU was administered in combination with vardenafil (22.4 mg/kg) every three days. For xenograft model of 5-FU-resistant CRC cells, 5×10^6^ HCT-8/5-FU cells were subcutaneously implanted into nude mice. Mice were administered with 5-FU (5 mg/kg) either alone or combined with Thiostrepton (17 mg/kg) every three days. All mice were sacrificed after 35 days, and the tumor tissues were used for subsequent studies.

### Cell lines and reagents

Human CRC cell lines RKO, HCT-116, HT-29, LOVO, SW620, SW480 and HT-29 5-FU resistant CRC cell lines (HT-29/5-FU) were cultured in Dulbecco's Modified Eagle's medium (GIBCO, USA) supplemented with 10% fetal bovine serum (GIBCO, USA). HCT-8 5-FU-resistant CRC cell lines (HCT-8/5-FU) and parental cells were cultured in RMPI 1640 medium with 10% fetal bovine serum. 5-FU was added in the medium of HCT-8/5-FU and HT-29/5-FU cells at a final concentration of 10 μg/ml and 1 μg/ml respectively. Thiostrepton and vardenafil were puchased from Selleck Chemicals.

### Colony formation assay

Single cell suspensions were seeded into 6-well plates at 300 cells/well. After 14 days of culture, cells were fixed with 4% formaldehyde and stained with Giemsa solution. The number of visible colonies was calculated. Each experiment was performed in triplicate.

### *In vitro* chemosensitivity assay

The IC50 values of cells were measured by Cell Counting Kit-8 assay (Dojindo Molecular Technologies). Single cell suspensions were dispersed in 96-well plates at a density of 5000 cells/well, and subjected to indicated treatment. After incubation at 37°C for 72 h, cells were incubated for another 2h with CCK8 reagent, followed by the detection of 450 nm absorbance using a microplate reader (Bio-Rad, Model 680).

### Flow cytometry

Apoptosis was measured by Annexin V-fluorescein isothiocyanate (FITC) apoptosis detection kit (Oncogene Research Products, Boston, MA) according to manufacturer's instruction. All of the analysis was performed in triplicate.

### Immunohistochemistry

Tissue slides were deparaffinized, rehydrated, followed by antigen retrieval. After the incubation of primary and secondary antibody, the slides were incubated with diaminobenzidine (DAB) (Dako, USA), and finally counterstained with hematoxylin (Sigma Chemical Co, USA). Primary antibodies are listed as follows: Ki67 (1:500, Abcam), FOXM1 (1:100, Santa Cruz Biotechnology), ABCC10 (1:25, Santa Cruz Biotechnology)

### Western blot

Total cell lysates were collected and protein concentration was measured. Equal amount of proteins was separated by SDS-PAGE and transferred onto polyvinylidene fluoride (PVDF) membranes (Millipore, USA). The membranes were blocked with 5% bovine serum albumin in TBST for 2h at room temperature and incubated with primary antibodies overnight at 4°C. Following the incubation with secondary antibodies at room temperature for 2h, proteins on the membrane were visualized with a chemiluminescence kit (Thermo Scientific). Primary antibodies are listed as follows: β-actin (1:1000, Cell Signaling Technology), FOXM1 (1:100, Santa Cruz Biotechnology), cleaved caspase-3 (1:1000, Cell Signaling Technology), cleaved caspase-7 (1:1000, Cell Signaling Technology), cleaved PARP (1:1000, Cell Signaling Technology) and ABCC10 (1:50, Santa Cruz Biotechnology).

### Quantitative reverse-transcription polymerase chain reaction (qRT-PCR)

Total RNA was extracted from tissues and cells with TRIzol reagent (Takara, Japan) according to manufacturer's instruction. Reverse transcription was conducted using the PrimeScript RT Reagent Kit (Takara). Real-time quantitative PCR was performed on triplicate samples in a reaction mix of SYBR Green (Takara) with ViiA 7 Dx Real-Time PCR System (Applied Biosystems). The mRNA levels were normalized against β-actin. Sequences of primers used for qRT-PCR in this study were listed in Supplementary Table 3.

### Chromatin immunoprecipitation (ChIP) assay

ChIP assay was performed with EZ-ChIP Kit (Millipore). Chromatin was immunoprecipitated with FOXM1 antibody and analyzed by qPCR. Sequences of primers for ChIP-qPCR were listed as follows: 5’-GGGAAATGTGGGGAA-3’ and 5’-AGAAGA CGGAACCTTA-3’; 5’-CTGCTGACCTTCCCTC-3’ and 5’-TAGTTGTAATTGTCTTCA-3’.

### Statistical analysis

All statistical analyses were performed with SPSS 20. 0 software package (SPSS, Chicago, IL, USA). Data was presented as mean ± SD with at least three independent experiments. Two-tailed Student's t test was used for comparison of means between two groups. Multiple group comparisons were analyzed with one-way ANOVA. P < 0.05 was considered statistically significant.

## SUPPLEMENTARY MATERIALS FIGURES AND TABLES







## Abbreviations

CRC: colorectal cancer
siRNA: small interfering RNA
5-FU: 5-Fluorouracil
shRNA: short hairpin RNA
PARP: poly ADP-ribose polymerase
ABCC10: ATP binding cassette subfamily C member 10
