# Supplementary material for: FOXM1 evokes 5-fluorouracil resistance in colorectal cancer depending on ABCC10
Source: Oncotarget. 2016 Dec 29;8(5):8574–89. doi: 10.18632/oncotarget.14351 (PMC5352423; doi:10.18632/oncotarget.14351)
Supplement: Supplementary file 1 [file oncotarget-08-8574-s001.pdf]

FOXM1 evokes 5-fluorouracil resistance in colorectal cancer depending on ABCC10

SUPPLEMENTARY FIGURES AND TABLES

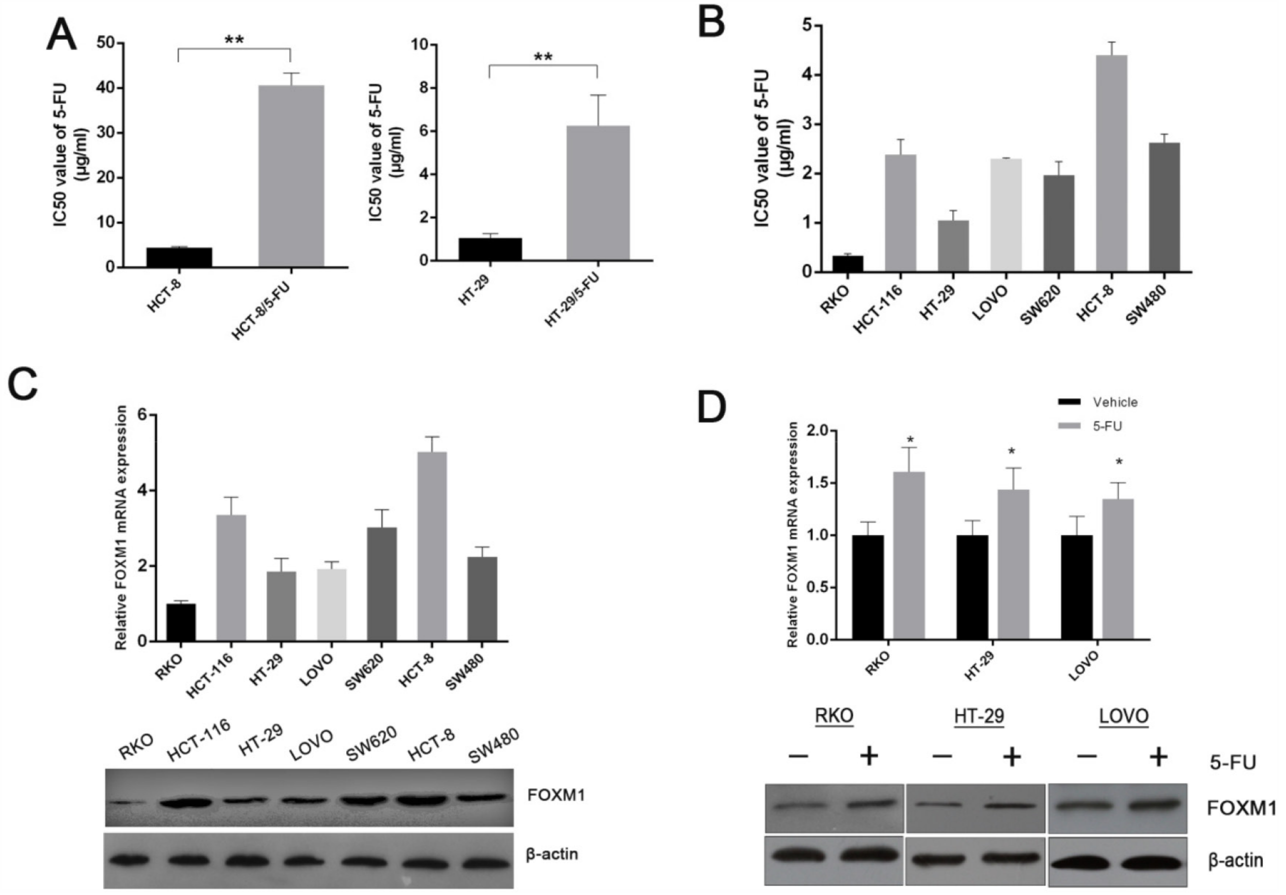

Supplementary Figure 1: A. IC50 values of 5-FU in HCT-8 and HCT-8/5-FU cells or HT-29 and HT-29/5-FU measured by CCK-8. **B.** IC50 values of 5-FU in colorectal cancer cell lines measured by CCK-8. **C.** qRT-PCR and Western blot assay of FOXM1 in colorectal cancer cell lines. **D.** qRT-PCR and Western blot assay of FOXM1 in RKO, HT-29, LOVO cell lines before and after 5-FU treatment.

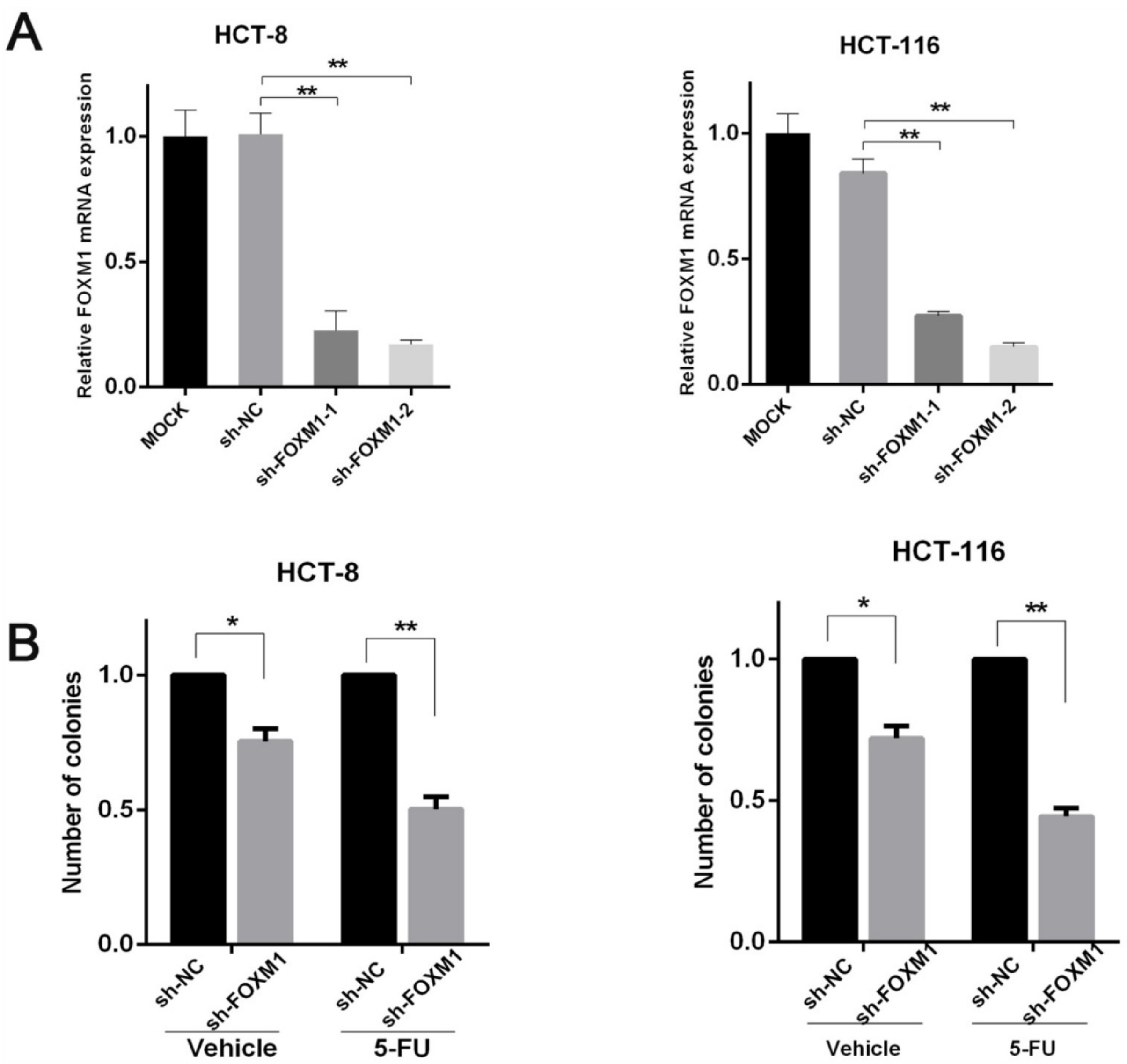

Supplementary Figure 2: A. qRT-PCR analysis of FOXM1 in knockdown and control CRC cell lines. **B.** Colony formation of FOXM1 knockdown and control CRC cells treated with 5-FU or not (n=3).

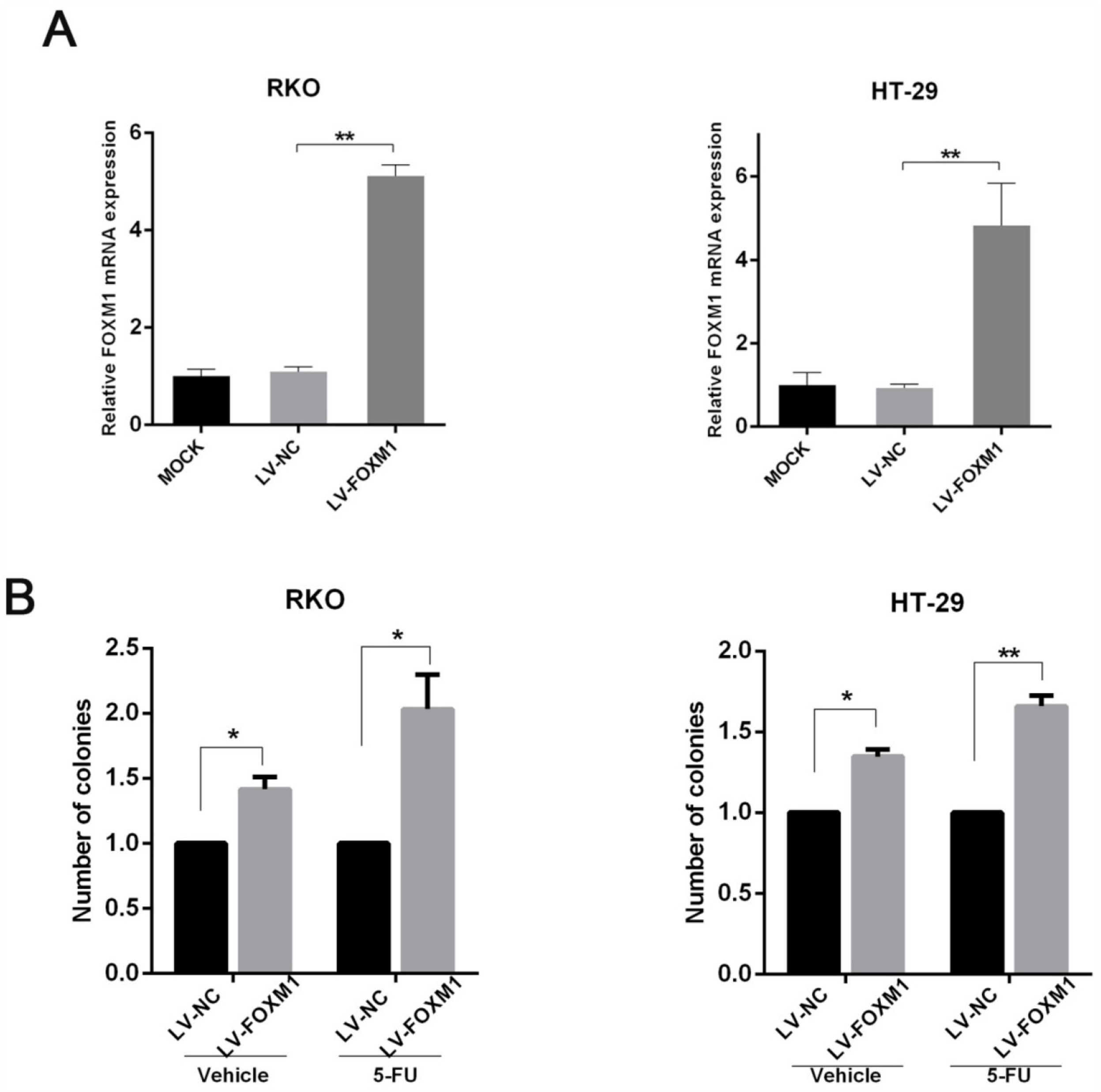

Supplementary Figure 3: A. qRT-PCR analysis of FOXM1 in overexpressing and control CRC cell lines. **B.** Colony formation of FOXM1 overexpression and control CRC cells treated with 5-FU or not (n=3).

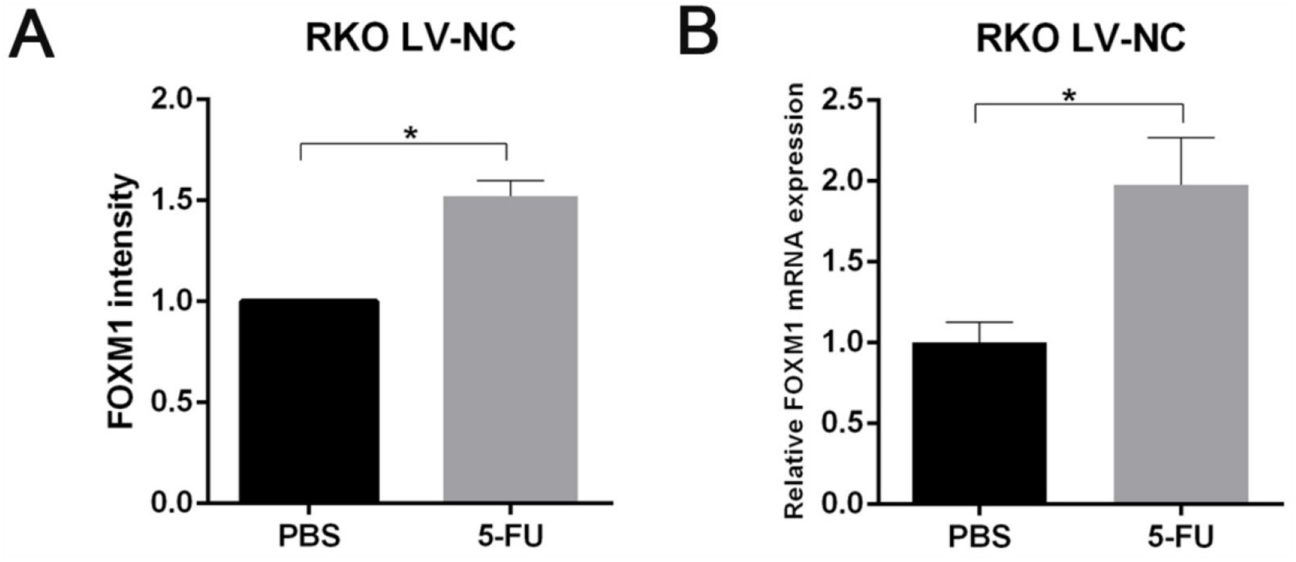

Supplementary Figure 4: A. Quantification of the IHC staining of FOXM1 in RKO LV-NC xenografts from mice treated with 5-FU or not (n=3). **B.** qRT-PCR analysis of FOXM1 in RKO LV-NC xenografts from mice treated with 5-FU or not (n=3).

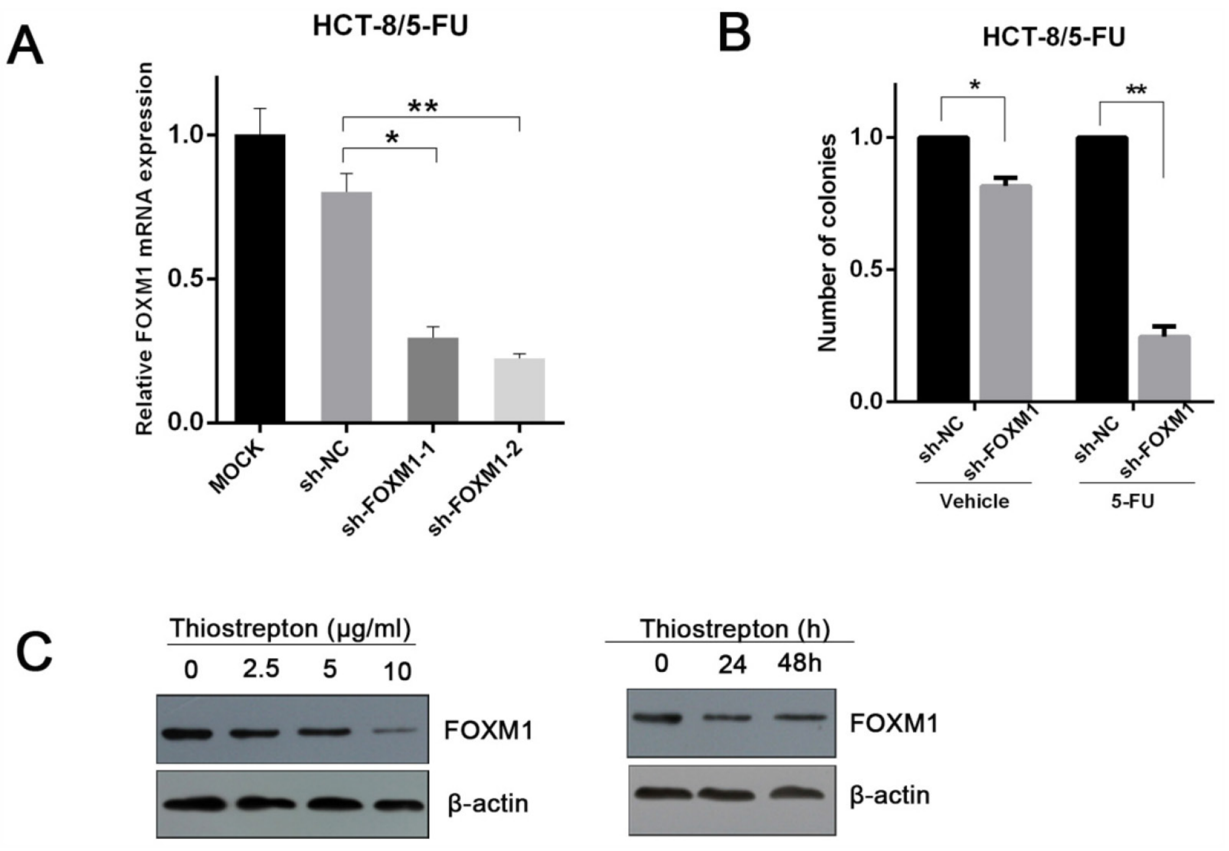

Supplementary Figure 5: A. qRT-PCR analysis of FOXM1 in knockdown and control HCT-8/5-FU cells. **B.** Colony formation of FOXM1 knockdown and control 5-FU resistant CRC cells treated with 5-FU or not (n=3). **C.** Western blot assay of FOXM1 in HCT-8/5-FU cells treated with thiostrepton with indicated concentrations or time.

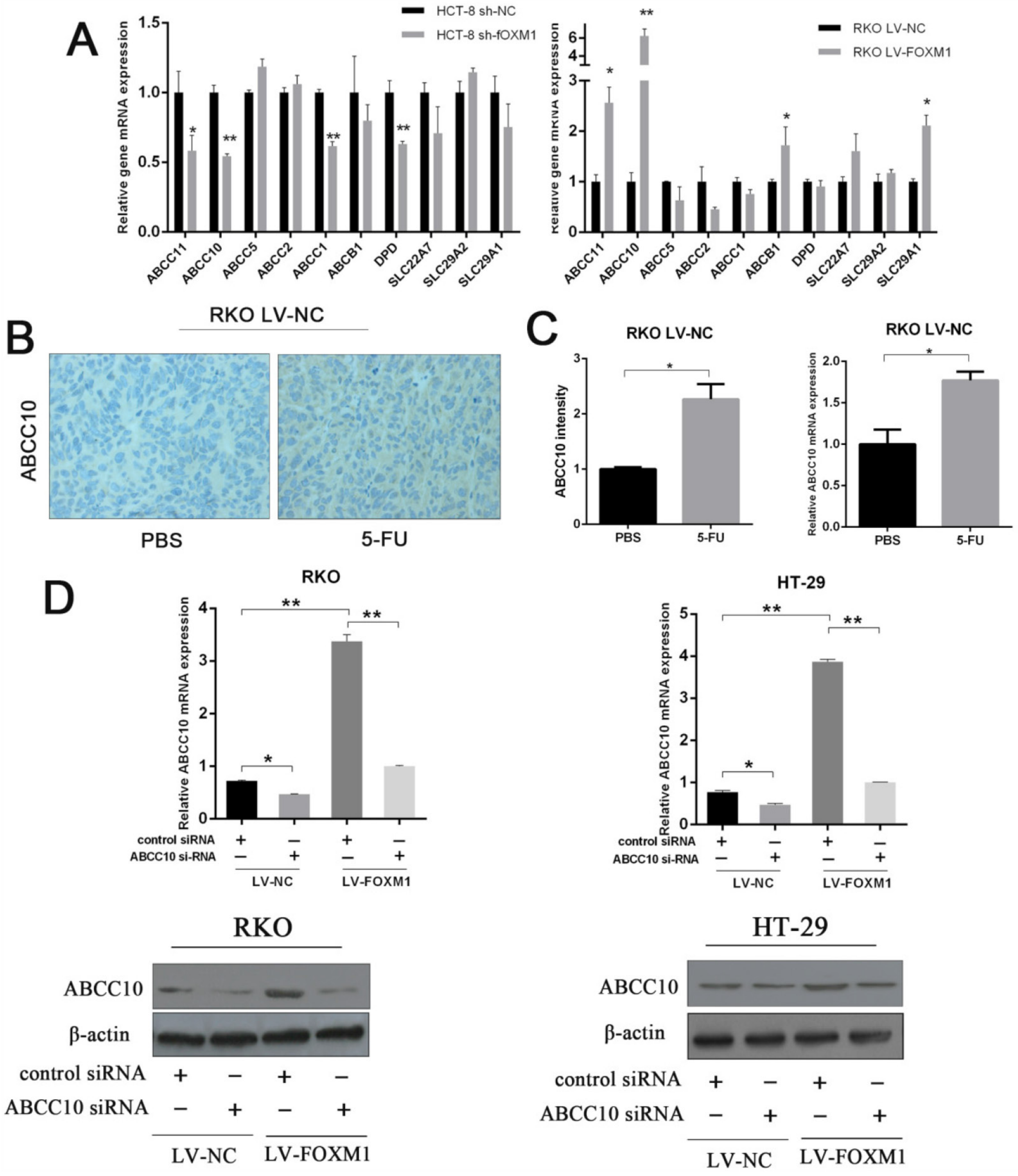

Supplementary Figure 6: A. qRT-PCR analysis of indicated genes in FOXM1 knockdown or overexpressing CRC cells. **B.** Representative immunostaining of ABCC10 in RKO LV-NC xenografts from mice treated with 5-FU or not (n=3). **C.** Quantification of the IHC stainings and qPCR levels of ABCC10 in RKO LV-NC xenografts from mice treated with 5-FU or not (n=3). **D.** qRT-PCR and Western blot analysis of ABCC10 level in FOXM1 overexpressing cells along with ABCC10 siRNA tranfection.

Supplementary Table 1: Gene expression values in resistant and sensitive CRC patients assessed by microarray

See Supplementary File 1

Supplementary Table 2: Sequences of shRNA and siRNA Against Specific Target

sh-FOXM1-1	5’-3’	GGCUGCACUAUCAACAAUATT
sh-FOXM1-2	5’-3’	CCAACAGGAGTCTAATCAA
sh-NC	5’-3’	TTCTCCGAACGTGTCACGT
si-ABCC10	Sense (5’-3’)	CCUUCCUGCUUUCCGUCUUTT
	Antisense (5’-3’)	AAGACGGAAAGCAGGAAGGTT
si-NC	Sense (5’-3’)	UUCUCCGAACGUGUCACGUTT
	Antisense (5’-3’)	ACGUGACACGUUCGGAGAATT

Supplementary Table 3: Sequences of PCR primers used in this study

See Supplementary File 2
