# Supplementary material for: FOXM1 evokes 5-fluorouracil resistance in colorectal cancer depending on ABCC10
Source: Oncotarget. 2016 Dec 29;8(5):8574–89. doi: 10.18632/oncotarget.14351 (PMC5352423; doi:10.18632/oncotarget.14351)
Supplement: Supplementary file 3 [file oncotarget-08-8574-s003.docx]

**Supplementary Table 3. Sequences of PCR primers used in this study.**

| SAA1 | Forward(5’-3’) | TCGTTCCTTGGCGAGGCTTTTG |
| --- | --- | --- |
|  | Reverse(5’-3’) | AGGTCCCCTTTTGGCAGCATCA |
| REG1A | Forward(5’-3’) | ACCAGCTCATACTTCATGCTGA |
|  | Reverse(5’-3’) | CCAGGTCTCACGGTCTTCAT |
| PHGDH | Forward(5’-3’) | CTGCGGAAAGTGCTCATCAGT |
|  | Reverse(5’-3’) | TGGCAGAGCGAACAATAAGGC |
| SERPINA1 | Forward(5’-3’) | ATGCTGCCCAGAAGACAGATA |
|  | Reverse(5’-3’) | CTGAAGGCGAACTCAGCCA |
| FOXM1 | Forward(5’-3’) | CGTCGGCCACTGATTCTCAAA |
|  | Reverse(5’-3’) | GGCAGGGGATCTCTTAGGTTC |
| DBH | Forward(5’-3’) | CCCTCCCCTATCACATCCCC |
|  | Reverse(5’-3’) | ACGGTCGGACATCCCAAAC |
| β-actin | Forward(5’-3’) | AATCGTGCGTGACATTAAGGAG |
|  | Reverse(5’-3’) | ACGTGTTGGCGTAACAGGTCTT |
| ABCB1 | Forward(5’-3’) | TTGCTGCTTACATTCAGGTTTCA |
|  | Reverse(5’-3’) | AGCCTATCTCCTGTCGCATTA |
| ABCC1 | Forward(5’-3’) | CTCTATCTCTCCCGACATGACC |
|  | Reverse(5’-3’) | AGCAGACGATCCACAGCAAAA |
| ABCC2 | Forward(5’-3’) | CCCTGCTGTTCGATATACCAATC |
|  | Reverse(5’-3’) | TCGAGAGAATCCAGAATAGGGAC |
| ABCC5 | Forward(5’-3’) | AGTCCTGGGTATAGAAGTGTGAG |
|  | Reverse(5’-3’) | ATTCCAACGGTCGAGTTCTCC |
| ABCC10 | Forward(5’-3’) | GTCCAGATTACATCCTACCCTGC |
|  | Reverse(5’-3’) | GCCAACACCTCTAGCCCTATG |
| ABCC11 | Forward(5’-3’) | GTGAATCGTGGCATCGACATA |
|  | Reverse(5’-3’) | GCTTGGGACGGAAGGGAATC |
| ABCC12 | Forward(5’-3’) | GGTGAAGGACCCTACCTTATCT |
|  | Reverse(5’-3’) | TTGGGTGCTAACCTTGCACA |
| SLC22A7 | Forward(5’-3’) | TTG TAC CCT ACG GTG CTC AG |
|  | Reverse(5’-3’) | CAC ACT CCA TCC AGC AAG G |
| SLC29A2 | Forward(5’-3’) | ATGAGAACGGGATTCCCAGTAG |
|  | Reverse(5’-3’) | GCTCTGATTCCGGCTCCTT |
| SLC29A1 | Forward(5’-3’) | TGAGCGGAACTCTCTCAGTG |
|  | Reverse(5’-3’) | TGAGGTAGGTGAATAACAGCAGG |
